# Primary care cohort study in the sequence of diagnosing chronic respiratory diseases and prescribing inhaled corticosteroids

**DOI:** 10.1038/s41533-018-0106-6

**Published:** 2018-10-09

**Authors:** Ilja Geraets, Tjard Schermer, Janwillem W. H. Kocks, Reinier Akkermans, Erik Bischoff, Lisette van den Bemt

**Affiliations:** 1 Department of Primary and Community Care, Radboud University Medical Center, Nijmegen, The Netherlands; 2 Netherlands Institute for Health Services Research NIVEL, Utrecht, The Netherlands; 3 Groningen Research Institute for Asthma and COPD, Department of General Practice and Elderly Care medicine, University of Groningen, University Medical Center Groningen, Groningen, The Netherlands; 4 Radboud Institute for Health Sciences, IQ Healthcare, Radboud University Medical Center, Nijmegen, The Netherlands

## Abstract

To prevent unnecessary use of inhaled corticosteroids (ICS), ICS treatment should only be started when the diagnostic process of asthma and COPD is completed. Little is known about the chronological order between these diagnoses and the start of ICS. We performed a retrospective cohort study, based on electronic medical records of 178 Dutch general practices, to explore the temporal relations between starting continuous use of ICS and receiving a diagnosis of asthma and/or COPD. The database included information of patients who were registered with a diagnosis of asthma and/or COPD in one of the practices during January 1, 2012 and December 31, 2013. Two or more successive prescriptions of ICS within 6 months were considered as continuous ICS treatment. The chronological order of events based on available dates were analysed using descriptive analyses. For 8507 patients with asthma, 4024 patients with COPD, and 801 patients with asthma–COPD overlap (ACO), the order of events could be analysed. In total, 1857 (14.4%) patients started ICS prior to their diagnosis, 11.5, 20.8, and 10.0% of patients with asthma, COPD, and ACO, respectively. In 53.4% of the patients, the first prescription of ICS was a combination inhaler with a long-acting bronchodilator. In this real-life primary care cohort, one in seven patients started ICS treatment prior to their diagnosis and approximately half of the patients started with a combination inhaler. Our findings suggest that there is relevant room for improvement in the pharmaceutical management of patients with these chronic respiratory diseases.

## Introduction

Asthma and chronic obstructive pulmonary disease (COPD) are prevalent chronic respiratory diseases in primary health care.^1,2^ Both diseases are frequently treated with inhaled corticosteroids (ICS). For several years now, ICS are listed in the top five of highest expenditure on medicines in the UK as well as in the Netherlands.^3,4^ In asthma, ICS treatment is the cornerstone of pharmacological management as it has beneficial effects on lung function, number of exacerbations, and quality of life.^5^ In COPD, the indication for ICS treatment is limited to patients with severe to very severe disease and frequent exacerbations, as it may reduce the number of exacerbations.^6,7^

In contrast with these indications, approximately half of the patients with COPD are treated with ICS,^8–10^ whereas in primary care only 10–20% of the patients seems to have frequent exacerbations.^11^ A recent study showed that even COPD patients with (very) severe airflow obstruction did not have more exacerbations after their ICS treatment was discontinued^12^ and there are indications that only patients with high blood eosinophil levels seem to benefit from continuation of ICS treatment.^13^ In addition, treatment with ICS can cause important side effects, like increased risks of pneumonia and osteoporosis.^6,14^

The Dutch asthma guidelines recommend to diagnose patients before prescribing ICS, because ICS can influence spirometric outcomes.^5,15^ However, in daily practice general practitioners (GPs) may start ICS at the moment a patient presents with bronchial complaints, before further diagnostic procedures are initiated or completed. Consequently, absence of airflow reversibility can either be the result of well-controlled asthma or of the absence of disease.^5^ Two Canadian studies showed that in one third of the patients with physician-diagnosed asthma, the diagnosis could not be objectified.^16,17^ Some of the patients might be labelled incorrectly as having asthma based on a good self-perceived symptomatic response to ICS and continue its use without an indication.

Another issue related to ICS treatment is the increasing use of combination inhalers (i.e. an inhaler that contains ICS plus a long-acting bronchodilator (LABA)). Approximately 40% of the patients with asthma and COPD use combination inhalers,^8^ whereas asthma guidelines recommend to start ICS treatment with a separate inhaler. Only when the intended treatment effects are not achieved, switching to a combination inhaler is indicated.^5,15^

Little is known about the chronological order between diagnosing patients with asthma and/or COPD and the start of ICS in daily practice. Primary aim of the current study was to explore the chronological order of starting ICS maintenance treatment and receiving a diagnosis of asthma and/or COPD in patients managed in general practice. We also examined how often GPs choose to prescribe ICS in a combination inhaler when starting ICS maintenance treatment.

## Results

### Patient selection and ICS treatment

**Fig. 1:**
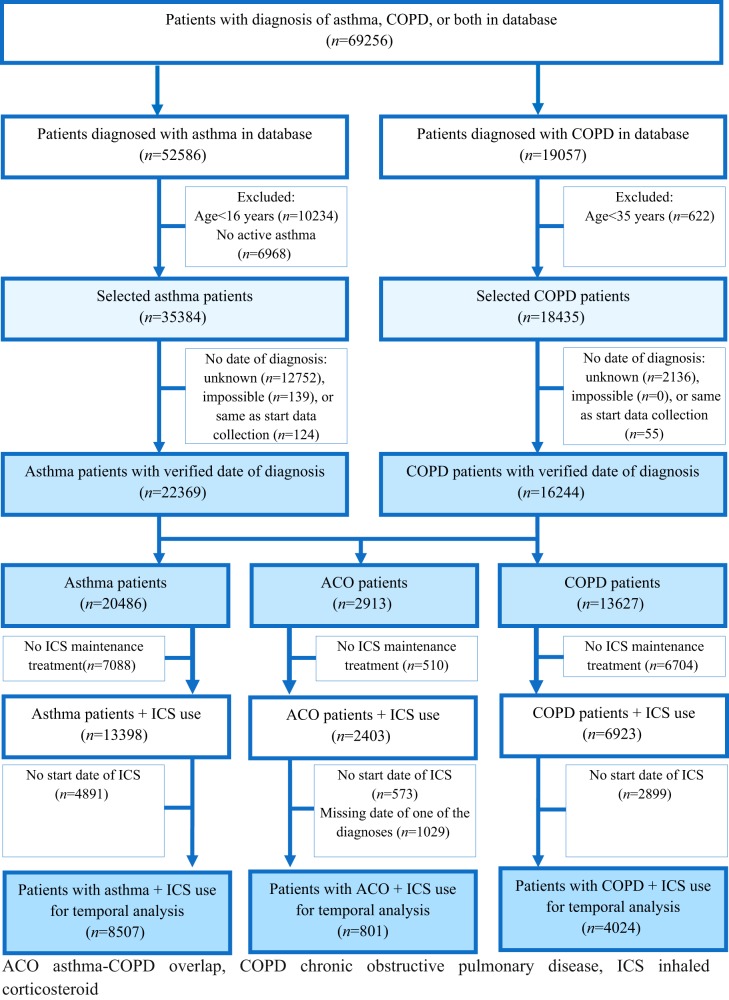
Flowchart of the selection procedure of patients with asthma and COPD in the database of the Department of Primary and Community Care at the Radboud University Medical Center, Nijmegen, the Netherlands

Table 1 shows characteristics of the study population. Of all patients with asthma in the database, 9.3% was diagnosed with COPD as well. Among patients with COPD, 17.2% was also diagnosed with asthma. About half (50.8%) of the COPD patients without concomitant asthma received ICS treatment. Verifiable dates of diagnosis could be found in 63.2% and 88.1% of the patients with asthma and COPD, respectively (Fig. 1). All relevant dates were available for 13,332 patients. This study population included 8507 patients who were diagnosed with asthma, 4024 patients diagnosed with COPD, and 801 patients with asthma–COPD overlap (ACO).

**Table 1:** Characteristics of patients in the cohort grouped by patients included in the analyses versus patients not included in the analyses

	Asthma^a^			COPD^a^		
	Total cohort (*n* = 35,384)	In analyses (*n* = 9,308)	Not in analyses^b^ (*n* = 26,076)	Total cohort (*n* = 19,057)	In analyses (*n* = 4,825)	Not in analyses^b^ (*n* = 14,232)
Male, *n* (%)	14,619 (41.3)	3597 (38.6)	11,022 (42.3)	10,072 (52.9)	2405 (49.8)	7667 (53.9)
Asthma, *n* (%)				3286 (17.2)	801^c^ (16.6)	2485^c^ (17.5)
COPD, *n* (%)	3286 (9.3)	801 (8.6)	2485 (9.5)			
ICS, *n* (%)	20,737 (58.6)	9308 (100.0)	11,429 (43.8)	10,674 (56.0)	4825 (100.0)	5849 (41.1)
LABA single inhaler, *n* (%)	4533 (14.2)	1631 (17.5)	2902 (12.8)	4547 (28.2)	1418 (29.4)	3129 (27.7)
LAMA single inhaler, *n* (%)	5552 (17.4)	1842 (19.8)	3710 (16.4)	11,952 (74.2)	3573 (74.1)	8379^c^ (74.2)
LAMA/LABA combined inhaler, *n* (%)	582 (1.8)	162 (1.7)	420^c^ (1.9)	1298 (8.1)	414 (8.6)	884^c^ (7.8)

*COPD* chronic obstructive pulmonary disease

^a^All patients with a diagnosis including asthma–COPD overlap

^b^Missing values on use of LABA and/or LAMA of asthma (*n* = 3393) and COPD (*n* = 2939) compared between the two subgroups were statistically significant (*p* < 0.05)

^c^No statistical significant difference (*p* > 0.05). All other differences in patient characteristics are significant

### Chronological order of diagnoses and ICS treatment

**Fig. 2:**
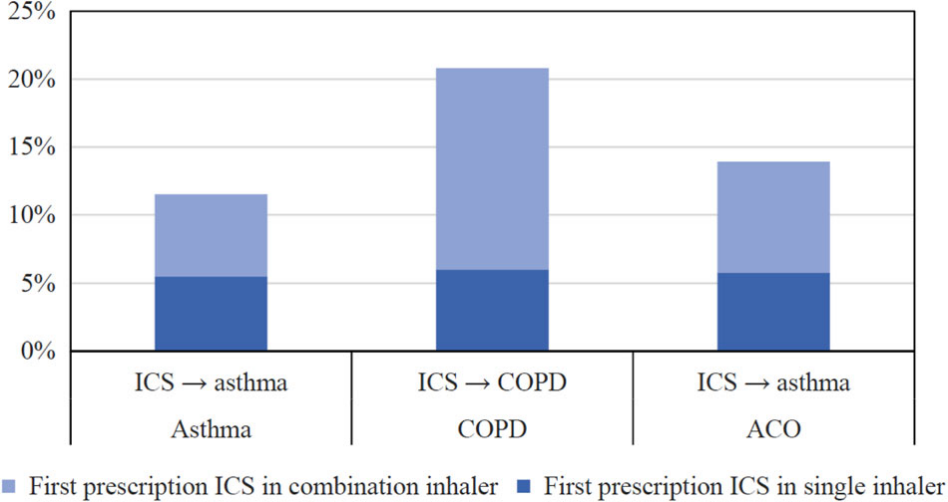
Percentages of patients with asthma, COPD, or ACO who used ICS before their (asthma) diagnosis and the percentages of combination inhalers (i.e. ICS plus long-acting bronchodilator) as first prescription

In total, 1898 (14.2%) patients started ICS treatment before a diagnosis of asthma and/or COPD had been established. Of the patients with asthma 11.5% started ICS prior to the diagnosis, in the patients with COPD this was 20.8%. Of the patients with ACO, 10.0% started ICS prior to both diagnoses. Table 2 shows the numbers and percentages per subgroup. Moreover, Fig. 2 shows the use of ICS as monotherapy and in a combination inhaler before their diagnoses were established.

**Table 2:** Number and percentages of chronological order of events per group of patients with the same diagnosis and the number and percentage of combination inhalers (i.e. ICS plus long-acting bronchodilator) as first prescription in patients with ICS maintenance treatment

	Chronological order of events			Total	Combination inhaler
	First event^a^	Second event^a^	Third event^a^	*n* (%)	*n* (% of total)
Asthma (*n* = 8,507)	Asthma	→ ICS		7525 (88.5)	3294 (43.8)
	ICS	→ Asthma		982 (11.5)	514 (52.3)
COPD (*n* = 4,024)	COPD	→ ICS		3188 (79.2)	2232 (70.0)
	ICS	→ COPD		836 (20.8)	598 (71.5)
ACO (*n* = 801)	Asthma	→ ICS	→ COPD	131 (16.4)	69 (52.7)
	Asthma	→ COPD	→ ICS	124 (15.5)	67 (54.0)
	COPD	→ ICS	→ Asthma	31 (3.9)	17 (54.8)
	COPD	→ Asthma	→ ICS	59 (7.4)	35 (59.3)
	ICS	→ Asthma	→ COPD	27 (3.4)	12 (44.4)
	ICS	→ COPD	→ Asthma	12 (1.5)	7 (58.3)
	Asthma/COPD	→ ICS		376 (46.9)	240 (63.8)
	ICS	→ Asthma/COPD		41 (5.1)	29 (70.7)
Total (*n* = 13,332)					7114 (53.4)

*ACO* asthma–COPD overlap, *COPD* chronic obstructive pulmonary disease, *ICS* inhaled corticosteroids

^a^Event: diagnosis of asthma ('asthma'), or diagnosis of COPD ('COPD'), or diagnosis of asthma and COPD ('asthma/COPD') on the same calendar date, or start of ICS treatment ('ICS')

### ICS combination inhaler as first prescription

Of all patients who were prescribed ICS for maintenance treatment, 53.4% started with a combination inhaler (Table 2). Of the patients with COPD, 14.9% started with a combination inhaler before the diagnosis COPD was established. The same applies for 6.0% of patients with asthma and 6.0% of the patients with ACO.

## Discussion

This study was performed to provide insight in the chronological order of events regarding the start of ICS treatment and a diagnosis of asthma and/or COPD in primary care patients. The main findings of this study are that approximately one out of seven patients started with ICS prior to a diagnosis and that more than half of the patients started with a combination inhaler instead of ICS in a separate inhaler.

The use of ICS among COPD patients in our study was comparable to percentages reported in the literature about COPD patients in the Netherlands and UK.^8,10^ A previous study found that 10–20% of COPD patients have frequent exacerbations.^11^ This may be lower in our study population, since a study based on the same database found that approximately 6% of the COPD patients had ≥2 exacerbations per year.^18^ Since 50.8% of the COPD patients without comorbid asthma in our study used ICS, there seems to be a significant overprescription.

The use of ICS among patients with asthma in our study (58.6%) was lower than found in literature (71%).^8^ This difference is probably explained by the restriction of continuous ICS use in our study, while other studies report on the number of patients with at least one prescription of ICS.^8^

The prevalence of ACO in our study is relatively low (9.3% and 17.2% of asthma and COPD patients respectively), compared to the assumed prevalence in literature (12–61%).^15,19^ However, a recent study assessed the impact of different ACO definitions on its prevalence and found that, when ACO was based on recorded diagnoses in primary care, like in our study, the prevalence was comparable (10.3%).^20^

In contrast with the recommendations in the Dutch COPD and asthma guidelines for GPs, approximately half of the patients who started with ICS treatment started with a combination inhaler instead of ICS alone. In a recent Dutch study, GPs mentioned that they regularly skip treatment steps, in particular in patients with severe symptoms. Moreover, GPs mentioned that they prescribe a combination inhaler when patients have an indication for LABAs, in order to improve the usability and compliance of ICS.^8,21^

### Strengths and limitations

A particular strength of this study is the use of a database with almost 70,000 patients with an asthma and/or COPD diagnosis from a primary care setting. This provides a non-selected, real-life insight in disease management decisions by GPs in daily practice. Moreover, in our study we focussed on continuous use of ICS. GPs may prescribe patients ICS for other reasons than asthma or COPD, and by looking at continuous use only, we excluded these prescriptions in our study.^22^

However, temporal relations could only be established for patients when all relevant dates of events were available and verifiable. Many patients had to be excluded owing to missing dates regarding either their diagnosis or their first ICS prescription. Patients who were excluded for the analysis of the chronological order of events showed statistically significant differences in sex and prevalence of ACO, although these differences were small. Therefore, it is likely that we introduced selection bias to our study by this unavoidable selection of patients with verifiable dates. Moreover, we were unable to compare the age of the patients at the moment of diagnosis. The study results only applied for adult asthma patients (≥16 years), and by excluding patients who were diagnosed with COPD before 35 years, some actual cases of COPD at a young age could be excluded incorrectly (for example, heavy smokers, other exposures, or alpha 1 antitrypsin deficiency).

We had to rely on the diagnostic codes in the medical record, which could be wrong. As with other routine care database studies, other relevant information like lung function and smoking status—that could justify or refute diagnostic conclusions—was unavailable and information on health-care utilization (like ICS prescription) was only available for a limited period. Therefore, we could not verify diagnoses based on objective lung function outcome, which is an important limitation. Moreover, a reintroduction of ICS in a period of more symptoms could be misinterpreted as a first prescription (if previous prescriptions were prior to the data collection period). We considered two courses of ICS within 6 months always as continuous ICS use as the number of prescribed inhalers was unknown. Therefore, two short courses of ICS within 6 months could be considered wrongfully as continuous ICS use. However, we believe that this problem did not frequently occur as the majority of the population had more than two prescriptions of ICS (94%). We also had to rely on data available in the database that may not always be complete (e.g. respiratory visits and diagnoses will not always be documented) or accurate. Therefore, it could be that the patient had used ICS for a longer period in the past and this was not documented in the current electronic medical record. Moreover, we did not analyse the exact time elapsed between the start of ICS treatment and the date of diagnosis. This could be relevant, as the maximum effect of ICS is achieved after several weeks and will not hamper diagnostic procedures for asthma in the first period a patient uses ICS.

### Implications for practice and research

Approximately one in seven patients used ICS prior to the diagnosis of asthma and/or COPD. Remarkable is the difference between asthma and COPD patients, 11.5% of the asthma patients started ICS treatment prior to the diagnosis, while in patients with COPD this was 20.8%. One possible explanation is that asthma is usually diagnosed at a younger age than COPD and GPs might be more conservative in prescribing medication in younger patients. Another explanation could be that the first presentation of patients with COPD is often during an exacerbation. In these patients, a first treatment of oral and ICS is started before further diagnostic procedures are done. In contrast to the NICE guideline, the Dutch primary care guideline on asthma recommend to start ICS after the diagnostic process is completed. Since ICS can suppress the bronchial inflammation and reduce reversibility of airway obstruction, a diagnosis of asthma is hard to objectify once ICS treatment has been started. This makes it difficult to distinguish between asthma and healthy airways.^23^ Our results show that many patients were labelled with a chronic respiratory disease after starting ICS treatment. There may be medical reasons to start ICS before the diagnosis is made; however, in these patients withdrawal of ICS should be considered. The findings that more than half of the COPD patients use ICS without having comorbid asthma and that one in every two patients started with a combination inhaler shows that GPs often make different choices than their COPD and asthma guidelines recommend. To validate our findings, further research should include information about the patients’ medical history and actual diagnostic test results and should analyse in more depth the exact time between the start of ICS and the date of diagnoses. Moreover, it would be interesting to compare outcomes between different settings as recommendations on the start of ICS use differ between countries and guidelines.

This study provides a first insight in the order of events regarding the start of continuous treatment with ICS and receiving a diagnosis of asthma and COPD in primary care. Our findings show that one in seven patients first start ICS maintenance treatment and are labelled with a diagnosis of asthma or COPD afterwards. Furthermore, in contrast with guideline recommendations, more than half of the patients were prescribed a combination inhaler instead of ICS in a separate inhaler, sometimes even before asthma and/or COPD was diagnosed. These findings suggest there is relevant room for improvement in the pharmaceutical management of patients with a chronic respiratory disease.

## Methods

### Design, setting, and data

We performed a retrospective cohort study based on data extracted from electronic medical records of 178 general practices in the eastern part of the Netherlands. In the Netherlands, all inhabitants are listed with a general practice that keeps an individual electronic medical record of registered patients. This record holds an overview of all diagnoses that the patient received, including information on outpatient and hospital care. Data are prospectively recorded and information on health-care utilisation is available for the period that the general practice maintains a medical record for the patient in the practice’s electronic patient journal system. For the current study, electronic medical record data of 69,256 patients who were registered in the participating practices during January 1, 2012 and December 31, 2013 and had a diagnosis of asthma and/or COPD were de-identified and extracted from the practices by the department of Primary and Community Care at the Radboud University Medical Center. For each patient, the following data were extracted: year of birth, sex, medical diagnoses based on International Classification of Primary Care (ICPC) coding, all contacts with the general practice, and all prescribed medication based on the Anatomical Therapeutic Chemical classification system.^24,25^ For our study, medical record and prescription data were available until March 1, 2017 or until a patient left the general practice or died. The recorded date of diagnosis was not always available in the data set. In that case, a patient was excluded for the analysis on chronological order of diagnoses and the start of ICS treatment. For this study, we used a database that contained only de-identified, anonymous information. Therefore, approval of an ethics committee was not required.

### Study population

Asthma patients were selected when they had an ICPC code R96(.01/02) before 2014 and were at least 16 years in 2014. To exclude patients who got the diagnosis asthma based on an incidental episode of respiratory complaints, we only included patients with at least one asthma-related contact or prescription for asthma medication in the previous 5 years (see Supplementary Information). We defined an asthma-related contact as a contact with the GP for dyspnoea (R02), wheezing (R03), and coughing (R05) based on ICPC code or a plain text notation in a recorded episode of asthma (R96). A diagnosis of COPD was defined as having received an ICPC code R95 before 2014 and after the age of 35 years.

### ICS and LABAs

All prescriptions of inhalation drugs that contain a corticosteroid (see Supplementary Information) were taken into account, including separate inhalers and combined ICS plus LABA inhalers. To exclude incidental ICS use, only two or more successive prescriptions of ICS within 6 months were considered as continuous ICS treatment. We categorized the choice of initial ICS treatment in combination inhalers and ICS in separate inhalers based on the first prescription. The use of separate LABA, long-acting muscarinic antagonist (LAMA) or combined inhalers (LABA/LAMA) was described.

### Sequence of diagnoses and start of ICS treatment

The chronological order of the start of ICS treatment and a diagnosis of asthma and/or COPD could be established if a known and verifiable date of the asthma diagnosis, the COPD diagnosis (or, when applicable, both these diagnoses), as well as a known date of the first ICS prescription were available. Reasons for missing verifiable dates of diagnosis were: no date recorded in the medical record, the recorded date of diagnosis was prior or equal to the date of birth, or the date of diagnosis was the same as the starting date of the patient’s electronic medical record. Medication prescription information was only available for the period that a patient’s electronic medical record was kept in the practice. In the Netherlands, ICS is usually prescribed for a period of 12 weeks, therefore we could not be sure that an ICS prescription in the first 14 weeks of medical record registration of a patient was the first prescription or a refill. We therefore excluded patients with a first recorded ICS prescription in the first 14 weeks, except when the respiratory diagnosis was clearly subsequent to the first ICS prescription. When the diagnosis of asthma and/or COPD was recorded on the same day as the first ICS prescription, we assumed that the treatment was started after the diagnosis.

### Statistical analysis

Analyses were performed with SPSS version 22.0. Patient characteristics were analysed using descriptive analyses and differences between subgroups using Chi-square tests. First, we described the number and percentages of patients with COPD, asthma, or ACO who used ICS and the proportion who used a combination inhaler. Next, the chronological order of asthma and/or COPD diagnoses and the first ICS prescription were analysed. Statistical significance was defined as *p* < 0.05.

## Electronic supplementary material

Supplementary information

## Data Availability

The data set generated and analysed during the study is currently not publicly available as the Radboud University Medical Center is developing a digital research environment where data will be made available in the near future (http://portal.umcn.nl/organisatie/im/Pages/Datastewardship.aspx). Until then, data can be made available by the corresponding author on reasonable request.

## Electronic supplementary material

**Supplementary Information** accompanies the paper on the *npj Primary Care Respiratory Medicine* website (10.1038/s41533-018-0106-6).
