# Supplementary material for: Primary care cohort study in the sequence of diagnosing chronic respiratory diseases and prescribing inhaled corticosteroids
Source: NPJ Prim Care Respir Med. 2018 Oct 9;28:37. doi: 10.1038/s41533-018-0106-6 (PMC6177428; doi:10.1038/s41533-018-0106-6)
Supplement: Supplementary file 1 — Supplementary information [file 41533_2018_106_MOESM1_ESM.docx]

**Supplementary Information**

Respiratory drugs categories in the database

| **Name** | **ATC** | **Contains corticosteroids** |
| --- | --- | --- |
| Salbutamol | R03AC02 |  |
| Terbutaline | R03AC03 |  |
| Salmeterol | R03AC12 |  |
| Formoterol | R03AC13 |  |
| Fenoterol | R03AK03 |  |
| Salbutamol/sodium cromoglicate | R03AK04 |  |
| Salmeterol/fluticasone | R03AK06 | Yes, combination inhaler |
| Formoterol/budesonide | R03AK07 | Yes, combination inhaler |
| Formoterol/beclometasone | R03AK08 | Yes, combination inhaler |
| Vilanterol/fluticasone furoate | R03AK10 | Yes, combination inhaler |
| Formoterol/fluticasone | R03AK11 | Yes, combination inhaler |
| Fenoterol/ipratropium bromide | R03AL01 |  |
| Beclometasone | R03BA01 | Yes |
| Budesonide | R03BA02 | Yes |
| Fluticasone | R03BA05 | Yes |
| Ciclesonide | R03BA08 | Yes |
| Ipratropium bromide | R03BB01 |  |
| Tiotropium bromide | R03BB04 |  |
| Cromoglicic acid | R03BC01 |  |
| Nedocromil | R03BC03 |  |
| Fenspiride | R03BX01 |  |
| Ephedrine | R03CA02 |  |
| Salbutamol (oral) | R03CC02 |  |
| Terbutaline (oral) | R03CC03 |  |
| Theophylline | R03DA04 |  |
| Montelukast | R03DC03 |  |
| Omalizumab | R03DX05 |  |
| Adrenergic inhalers | R03A |  |
